# Exploring the structural, mechanical, electronic, thermodynamic and thermoelectric properties of caesium based ABX_3_ perovskite CsOsX_3_ (X: Cl, Br)

**DOI:** 10.1039/d4ra04628e

**Published:** 2024-08-09

**Authors:** Sakshi Gautam, Dinesh C. Gupta

**Affiliations:** a Condensed Matter Theory Group, School of Studies in Physics, Jiwaji University Gwalior – 474001 MP India sakshi.parashartdl1234@gmail.com sosfizix@gmail.com

## Abstract

Here, we have investigated properties of caesium based halide perovskites with the help of density functional theory. We employed the generalized gradient approximation (GGA) functional to determine the structural characteristics. Conversely, for evaluating the electronic and thermoelectric properties of these materials we utilized the modified Becke and Johnson (mBJ) potential functional. Our findings indicate that these materials exhibit semiconducting properties. Furthermore, our analysis of the transport properties using the Boltzmann transport equation indicates that the studied perovskites are well-suited for thermoelectric applications.

## Abstract 2

Here, we have investigated properties of caesium based halide perovskites with the help of density functional theory.

## Graphical abstract

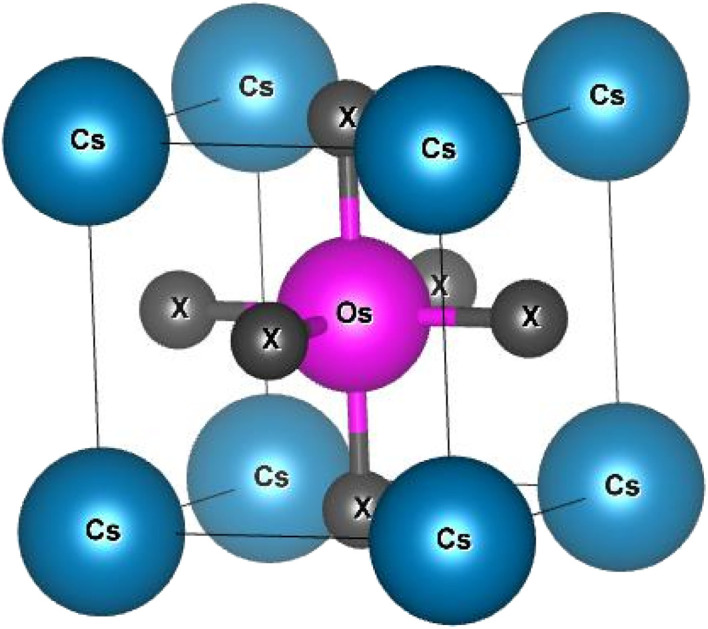

## 1. Introduction

In the realm of semiconductor materials, perovskite-based compounds have emerged as a revolutionary class, offering unprecedented opportunities across various technological applications.^1–3^ Nonmagnetic semiconductor halide perovskites, in particular, have garnered significant attention due to their unique properties and promising potential in optoelectronics, thermoelectricity, photovoltaics, and light-emitting devices.^4,5^ Unlike traditional perovskites that often exhibit magnetic properties stemming from transition metal ions, nonmagnetic semiconductor halide perovskites, characterized by their ABX_3_ composition involving elements from groups 13 and 14 of the periodic table, offer intriguing prospects for both fundamental research and practical applications.^6–8^ The term “nonmagnetic semiconductor halide perovskite” encompasses a broad class of materials characterized by their nonmagnetic nature and halide-based composition. Recently, several halide-based perovskite materials like InSnX_3_, ASiF_3_, ASiCl_3_ and Cs_2_GeSnX_6_, have been reported as non-magnetic semiconductors and as suitable candidate for optoelectronics and thermoelectric fields.^9–12^ These materials present a rich playground for investigating the interplay between crystal structure, electronic properties, and device performance. Hence, through a literature survey of different materials we have studied different properties of two halide based CsOsX_3_ (X = Cl, Br) perovskite materials with the help of density functional theory. These alloys have not been extensively reported in existing literature, highlighting their novelty and potential for exploration. Because they have not been extensively reported, CsOsCl_3_ and CsOsBr_3_ present an opportunity to delve into uncharted territory. Exploration of these materials using density functional theory (DFT) allows for the prediction and understanding of their fundamental properties, including band structure, electronic states, optical transitions *etc.* Such exploration is crucial for identifying promising applications in fields such as optoelectronics, photovoltaics, and thermoelectric. The specific composition of CsOsX_3_ (where Cs is caesium, Os is osmium, and X can be various halide ions) lends itself to unique structural and electronic properties. This composition typically involves heavy elements like osmium, which can influence electronic band structures and carrier mobilities, potentially enhancing device performance. Their crystal structure and electronic properties also make CsOsX_3_ materials viable candidates for thermoelectric applications. The ability to convert waste heat into electricity relies on materials with high electrical conductivity and low thermal conductivity, characteristics that can be tailored in CsOsX_3_ perovskites. In summary, CsOsX_3_ perovskite materials stand out in the field due to their unique combination of nonmagnetic semiconductor behaviour, favourable electronic properties, and potential applications in optoelectronics, photovoltaics, and thermoelectric. Their study using density functional theory (DFT) aims to uncover deeper insights into their fundamental properties, paving the way for future advancements in semiconductor device technologies. The insights gained from this research not only contribute to the fundamental understanding of halide perovskite materials but also pave the way for the design and engineering of next-generation semiconductor devices with enhanced performance and functionality. By bridging the gap between theory and experiment, our study aims to accelerate the development of innovative technologies based on these intriguing materials.

## 2. Computational details

In this research, we conducted first-principle calculations to assess the structural, elastic, electronic and thermoelectric properties of CsOsX_3_ (where X = Cl, Br) perovskites. These calculations were carried out using Density Functional Theory (DFT) implemented in the WEIN2K. We employed the Generalized Gradient Approximation with the Perdew–Burke–Ernzerhof (GGA-PBE) functional and modified Becke–Johnson potential to treat the exchange correlation functional.^13,14^ In multi-electron systems, the total wavefunction is often expressed using various basis sets. To determine the cut-off parameter *R*_MT_*K*_Max_ = 7.0 was used. For the Self-Consistent Field (SCF) calculation, we employed a 10 × 10 × 10 *k*-point grid. Additionally, we utilized the elastic package ingrained in WEIN2K to calculate elastic properties of the perovskites.^15^ For characterizing the thermal properties, the Gibbs2 package,^16^ integrated into Wien2k, was employed. Furthermore, to analyse the thermoelectric properties of these alloys, the semi-classical Boltzmann theory-based Boltz Trap code was interfaced with the WIEN2K code.^17^

## 3. Results and discussions

The following sections outline the results and discussions regarding the structural, mechanical, electronic, thermodynamic, and thermoelectric properties of the CsOsX_3_ perovskites.

### 3.1. Structural properties

**Fig. 1:**
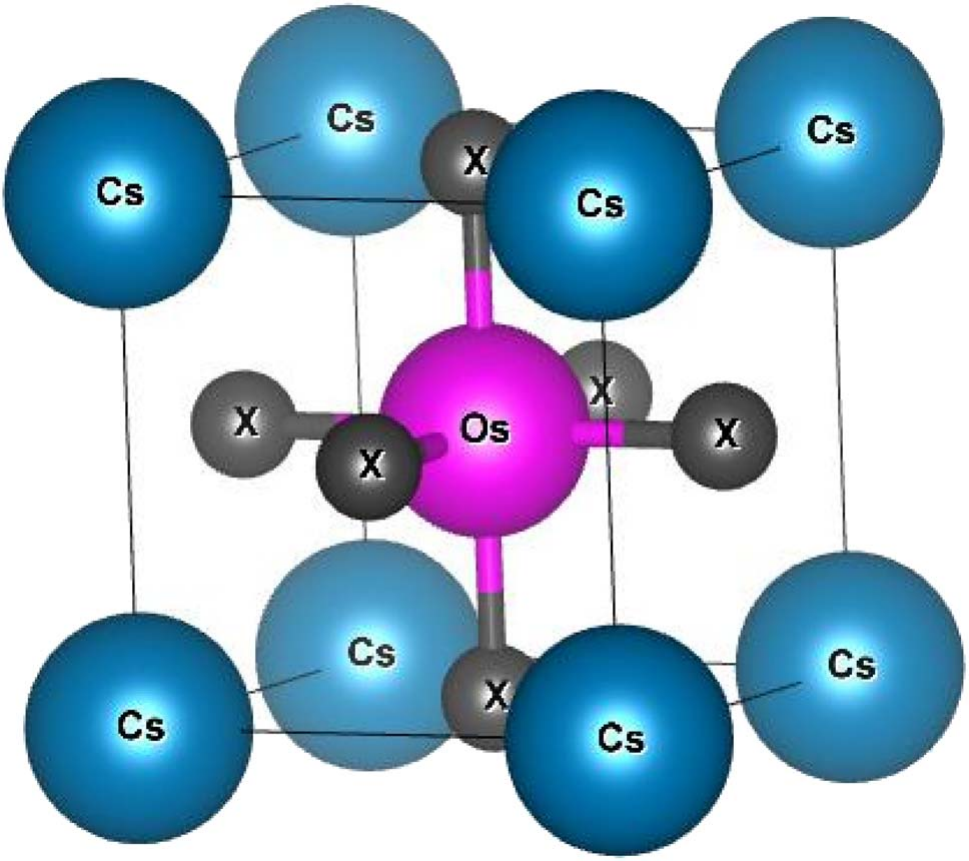
Crystal structure of CsOsX_3_ (X = Cl, Br) alloys.

**Fig. 2:**
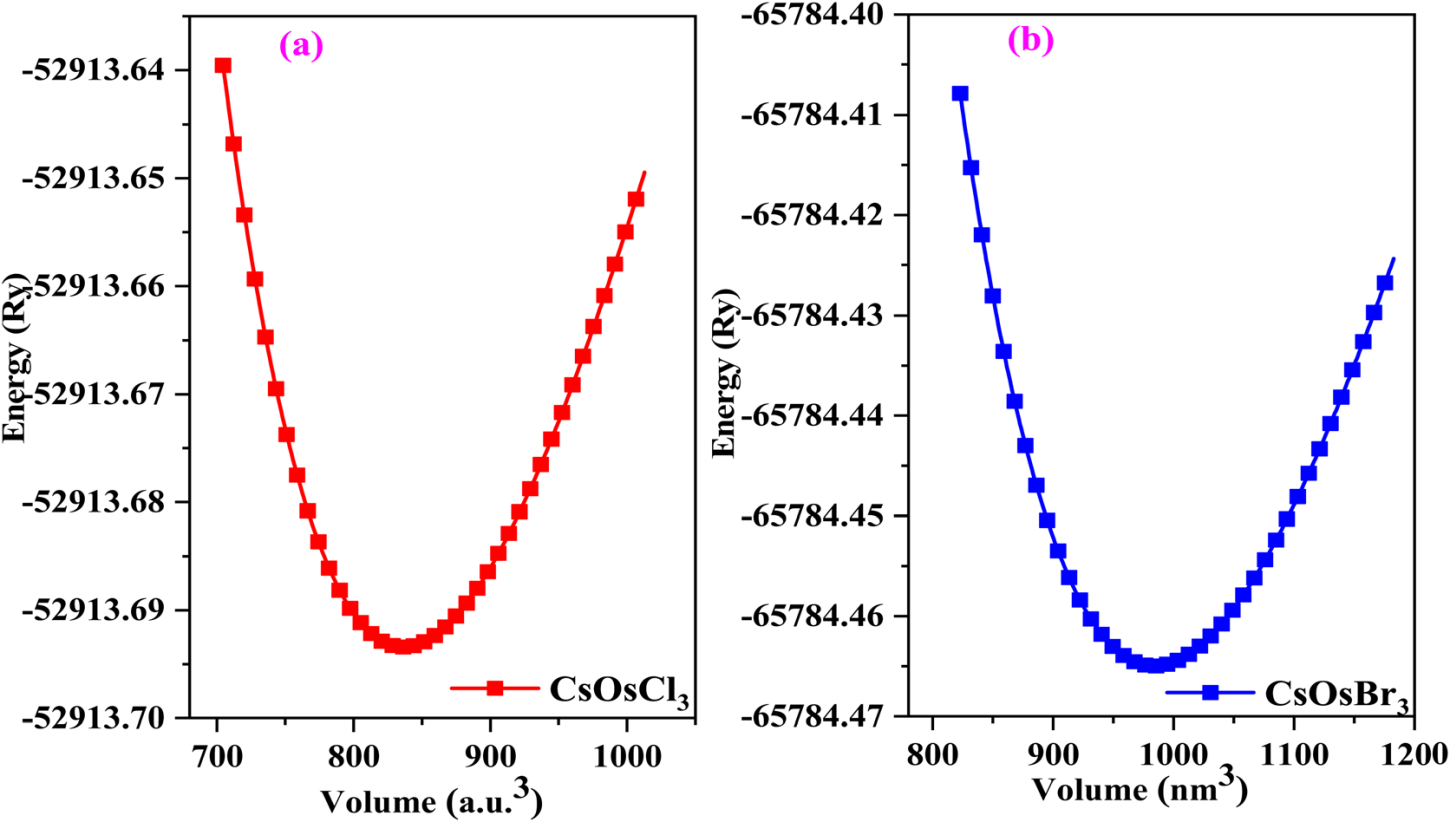
(a and b) Optimization plot of CsOsX_3_ (X = Cl, Br) alloys.

Generally, ABX_3_ type compounds typically feature a cubic crystal structure, with the A-site occupied by a large cation, the B-site by a transition or inner-transition element and the X site by anion. In the unit cell, the positions of atoms in the CsOsX_3_ crystal structure are as follows Cs atom is located at the corners, the Os atom occupy the body center, and the X atoms are positioned at the face centres. These atomic arrangements correspond to the Wyckoff positions 1a (0, 0, 0) for Cs, 1b (0.5, 0.5, 0.5) for Os, and 3c (0.5, 0.5, 0) for X, respectively. The optimized crystal structure, depicting these crystallographic sites, is presented in Fig. 1. Next, to start the simulation we have find the lattice constants of the given alloys with the help of relation enumerated as: *a*_0_ = *α* + *β*(*r*_A_ + *r*_X_) + *γ*(*r*_B_ + *r*_X_) here, *α* = 0.06741, *β* = 0.4905, and *γ* = 1.2921 are constants and *r*_A_ and *r*_B_ are radius of cation and *r*_X_ is radius of anion.^18^ Continuing, the current materials were then simulated in ferromagnetic (FM) and non-magnetic (NM) configurations using analytical lattice constants. Both the materials have minimum energy in NM phase hence, these materials exhibit non-magnetic behaviour. The optimization curve in non-magnetic phase is pictured in Fig. 2. From here we extract the ground state structural parameters by conducting a least-squares fitting of the crystal energy against the unit cell volume. This fitting was carried out using the Birch–Murnaghan equation of state.^19^ All the obtained parameters are listed in Table 1.

**Table 1:** Calculated lattice parameters (*a*_o_), minimum free energy (*E*_0_), bulk modulus (*B*), derivative of bulk modulus (*B*′), volume (*V*), cohesive energy and formation energy for CsOsX_3_ alloys (X: Cl, Br)

Parameters	CsOsCl_3_	CsOsBr_3_
*a*_o_ (a.u.)	4.98	5.26
*E*_0_ (Ry) [NM]	−52 913.69	−65 784.46
*B* (GPa)	52.81	43.91
*B*′	5.46	5.27
Volume (a.u.^3^)	836.46	984.07
*E*_Coh_ (eV per atom)	2.12	1.60
Δ*H* (eV)	−0.76	−0.59

Further, we have computed cohesive energy which is the energy required to separate the material into its individual atoms. It measures the strength of bond. To design and develop materials for different applications it is a crucial parameter. The computed values of cohesive energy per atom of both the materials are listed in Table 1. Also, we have calculated formasive energy by using the relation:Δ*H*_f_ = *E*_total_ − *μ*_Cs_ − *μ*_Os_ − 3*μ*_X_where, *E*_total_ is the total energy of the alloy and *μ*_Cs_, *μ*_Os_ and *μ*_X_ are the chemical potentials of Cs, Os and X, respectively. A negative value dictates the thermodynamically stability of the alloy. Here, for both the alloys the computed values are enlisted in Table 1 indicating the stability of the materials.^20,21^

### 3.2. Mechanical properties

The elastic constants play a crucial role in determining the mechanical behaviour, strength, and stability of a material. In order to thoroughly understand these properties, it's essential to express the elastic constants comprehensively. In materials exhibiting cubic symmetry, the complexity of determining elastic constants simplifies significantly. Despite the potential intricacy of the material's structure, the elastic constants consolidate into just three independent parameters *i.e. C*_11_, *C*_12_, *C*_44_.^22^ These constants are pivotal as they capture the material's reaction to stress in distinct directions, offering vital insights into its mechanical properties. A profound comprehension of these constants is essential for forecasting the material's behaviour under diverse mechanical scenarios, facilitating the design and enhancement of materials tailored for particular applications. The computed values of these constants are detailed in Table 2. Further, the Born–Huang stability criteria is followed.^23^ Using these constants, we've computed several other elastic parameters, including Young's modulus, bulk modulus, shear modulus, and more which are comprehensively outlined in Table 2. We calculate the bulk and shear moduli by Voigt–Reuss–Hill method.^24^ Subsequently, we determined another crucial property Young's modulus. Furthermore, we derived additional parameters such as Poisson's ratio to provide a comprehensive characterization of the material's mechanical behaviour. Furthermore, the Zener's anisotropy factor, calculated which clearly indicates the anisotropic nature of both the perovskites.^25^ Additionally, the computed values of Pugh's ratio and Cauchy pressure values delineate the ductile nature of the materials.^26^ Given the anisotropic nature of these materials' elastic waves exhibit different velocities in different directions. Consequently, we have determined the magnitude of longitudinal (*v*_l_) and transverse waves (*v*_t_1__ and *v*_t_2__) along (100), (110), (111) directions as enlisted in Table 3 by employing Bugger's relation.^27^ Moreover, we have computed Debye temperature *θ*_D_ with the help of mean sound velocity *v*_m_ calculated as: 
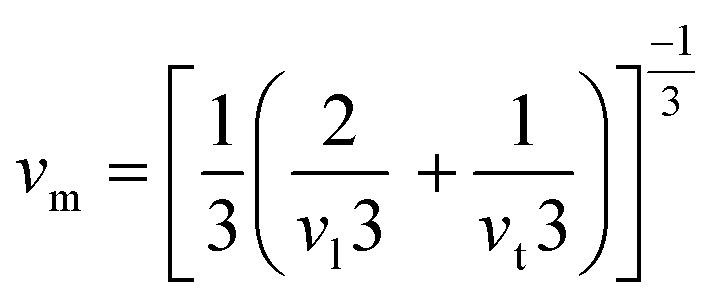
, where, *v*_l_ and *v*_t_ are longitudinal and transverse sound velocities which can be found using Navier's equation^28^ expressed as: 
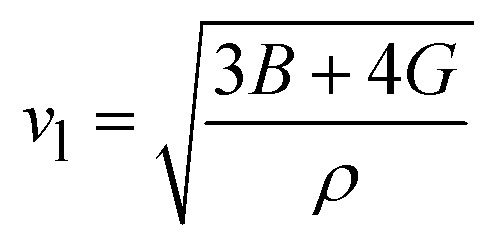
 and 
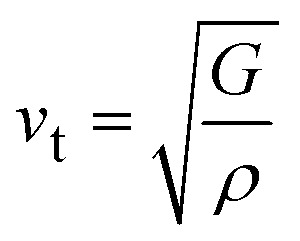
. The obtained values of longitudinal velocity, transverse velocity, mean velocity are mentioned in Table 4. Debye temperature *θ*_D_ can be calculated as 
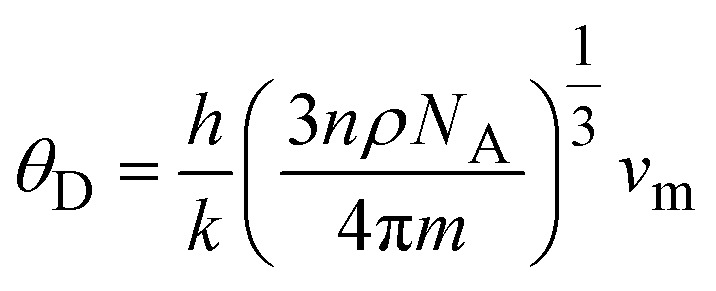
, where *h* is Plank's constant, *k* is Boltzmann's constant, *N*_A_ is Avogadro number, *ρ* is density and *v*_m_ is average sound velocity. The calculated values of *θ*_D_ for these alloys are also enlisted in Table 4.

**Table 2:** Calculated elasto-mechanical parameters *C*_11_, *C*_12_, *C*_44_, *G*, *B* and *Y* in (GPa)

Alloy	*C*_11_	*C*_12_	*C*_44_	*G*	*B*	*Y*	*N*	*A*	*B*/*G*	*C*′′
CsOsCl_3_	78.26	41.02	17.74	18.08	53.43	48.75	0.34	0.95	2.95	23.28
CsOsBr_3_	75.98	28.19	27.68	26.09	44.42	65.38	0.25	1.15	1.69	0.52

**Table 3:** Calculated sound (m s^−1^) and averaged velocities along different directions

Alloy	*v*_l_			*v*_t_1__			*v*_t_2__			*v*_m_		
Planes	[100]	[110]	[111]	[100]	[110]	[111]	[100]	[110]	[111]	[100]	[110]	[111]
CsOsCl_3_	1840	1830	1826	876	897	890	876	897	890	979	990	994
CsOsBr_3_	1719	1762	1775	1037	964	989	1037	964	989	1139	1103	1094

**Table 4:** The calculated longitudinal, transverse, mean velocities (m s^−1^) and Debye temperature (K)

Alloy	*v*_t_	*v*_l_	*v*_m_	*θ*_D_
CsOsCl_3_	884	1832	988	135
CsOsBr_3_	1007	1752	1111	142

#### Phonon stability

**Fig. 3:**
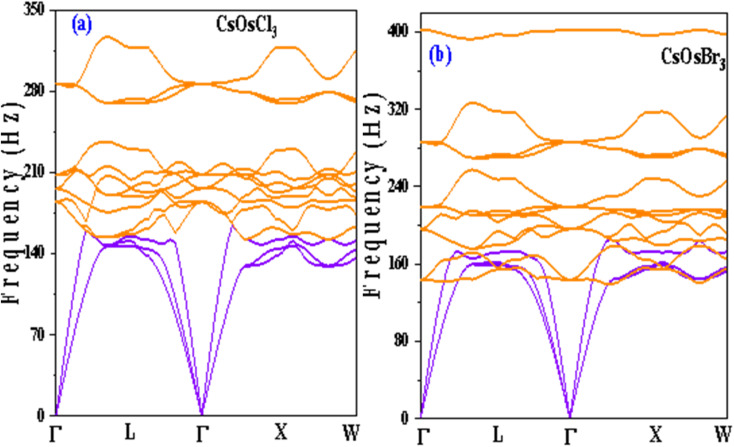
(a and b) Phonon dispersions along the high symmetric directions of Brillouin zone of CsOsX_3_ (X = Cl, Br) alloys.

Phonon dispersion diagrams are crucial for understanding the dynamic properties and vibrational characteristics of materials. In this study, density functional perturbation theory (DFPT) as implemented in Quantum ESPRESSO was used to assess the dynamical stability of the primitive unit cells of the studied perovskites. The analysis examined 15 phonon branches, as shown in Fig. 3(a) and (b). The absence of any negative frequencies in these phonon dispersion plots confirms the dynamical stability of these materials. Hence, we can say that these alloys are dynamically stable.

### 3.3. Electronic properties

**Fig. 4:**
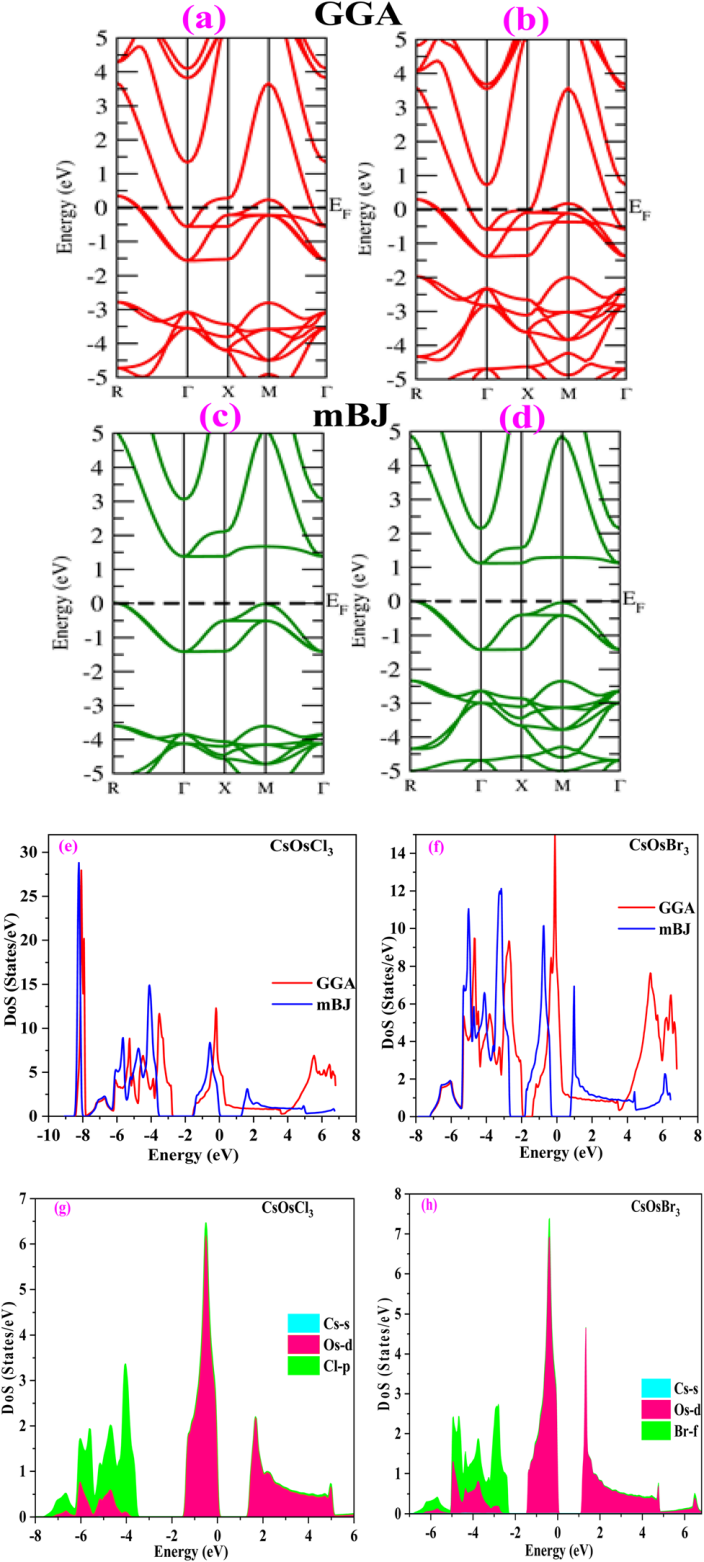
(a–h) Band structure by GGA (red colour), mBJ (green colour) approximation and density of states [total (e and f) and partial (g and h)] of CsOsX_3_ (X = Cl, Br) alloys.

To understand the electronic properties of materials, we have studied band structures and density of states of the titled alloys. At first we have computed the band structure which tells us about the energy levels of electrons in a material. The band structure of CsOsX_3_ alloys by GGA approximation is shown in Fig. 4(a) and (b). From the band structure we can see that these alloys retain the metallic nature. To improve the accuracy of electronic band structures we have employed mBJ potential. The band profile of both the materials by mBJ approximation is shown in Fig. 4(c) and (d). From the band structures we can see that energy bands are shifted from the Fermi level resulting in band gap between the valence and conduction band. The calculated band gaps are (1.36, 1.10) eV for CsOsCl_3_ and CsOsBr_3_ respectively. Hence, we can say that these two materials are semiconductor in nature. Additionally, the electronic band structure indicates that the halide perovskites being studied exhibit characteristics of direct bandgap semiconductors. This is evidenced by the conduction band minimum and valence band maximum aligning precisely at the *Γ*-point of the Brillouin zone.

Further, we have computed density of states (total and partial) for both the materials. The plot of total density of states is shown in Fig. 4(e) and (f) which also confirms the semiconducting nature of these materials. Also, to understand the contribution of each state we have computed partial density of states as shown in Fig. 4(g) and (h). From where, we can see there is a negligible contribution of Cs-s state. Due to the unique electronic properties these materials have notable applications in optoelectronic devices, power devices, photovoltaics, sensors *etc.*^29,30^

### 3.4. Thermodynamic properties

**Fig. 5:**
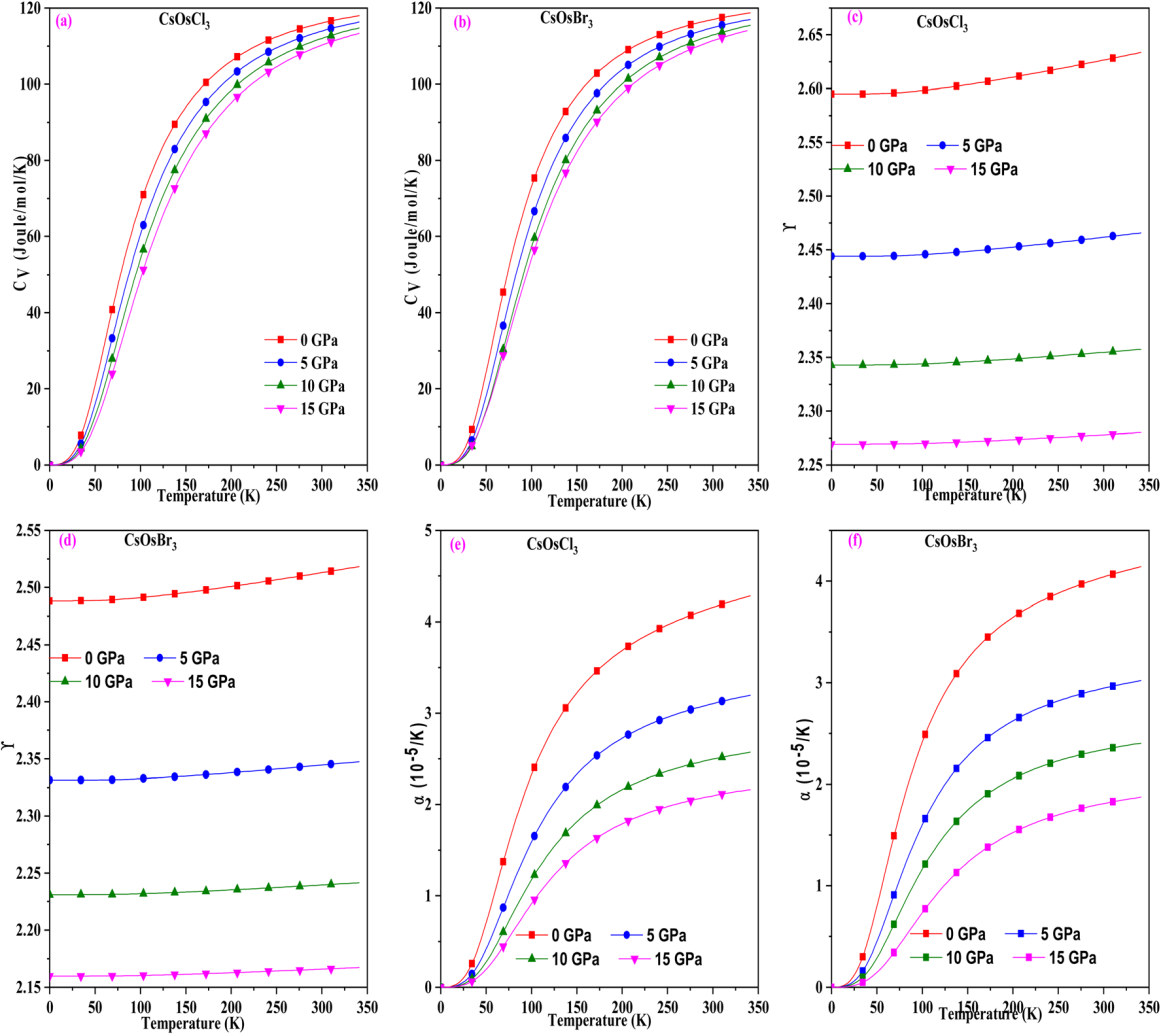
(a–f) The variation of specific heat, Gruneisen parameter and thermal expansion with temperature for CsOsX_3_ (X = Cl, Br) alloys.

To study the temperature and pressure effect on these materials we have computed different thermodynamic parameters. Initially, we calculated the specific heat capacity, which impacts thermal performance and energy transfer processes. The graph in Fig. 5(a) and (b) illustrates the variation for these perovskites, revealing that it increases with rising temperature and eventually stabilizes at higher temperatures, adhering to the Dulong–Petit law.^31^ The upward trend at lower temperatures is attributed to the heightened atomic vibrations accompanying temperature increases. Conversely, at elevated temperatures, molecules possess greater thermal energy and exhibit increased mobility. Consequently, the effectiveness of increasing average thermal energy diminishes. The specific heat capacity values for these materials at room temperature are (116.05 and 118.72) J mol^−1^ K^−1^ for CsOsCl_3_ and CsOsBr_3_ alloys respectively.

Subsequently, we computed the Gruneisen parameter (*γ*), a dimensionless quantity providing insight into the thermal state of the material and the variation of anharmonicity within the crystal lattice.^32^ For these materials, we observed a marginal increasing trend with rising temperature. However, notably, there is a substantial change with alterations in pressure, as illustrated in Fig. 5(c) and (d), suggesting that the pressure effect supersedes the temperature effect. The calculated values of the Gruneisen parameter (*γ*) are 2.62 and 2.51 for CsOsCl_3_ and CsOsBr_3_ alloys, respectively. Following that, we computed the thermal expansion coefficient (*α*), expressed as *α* = *γC*_V_/*B*_T_*V* to assess the extent of expansion possible. Fig. 5(e) and (f) depicts the variation of *α* at different pressures and temperatures. It's evident from this figure that *α* increases with temperature, as the bond strength decreases with temperature rise, leading to increased thermal expansion. Conversely, *α* decreases with increasing pressure, as pressure strengthens the bonding among atoms, thus tightly holding them together, resulting in decreased thermal expansion. The value of *α* at room temperature is (4.15 and 4.14) × 10^−5^ K^−1^ for CsOsCl_3_ and CsOsBr_3_ alloys respectively.

### 3.5. Thermoelectric properties

The evaluation of electric transport behaviour is crucial in comprehending the significance of a material's thermoelectric characteristics, as it directly influences the conversion of thermal energy into electrical energy. Here, to check the thermoelectric performance of these materials we have computed Seebeck coefficient (*S*), electrical conductivity (*σ*/*τ*), thermal conductivity (*κ*), power factor and figure of merit (*zT*).

**Fig. 6:**
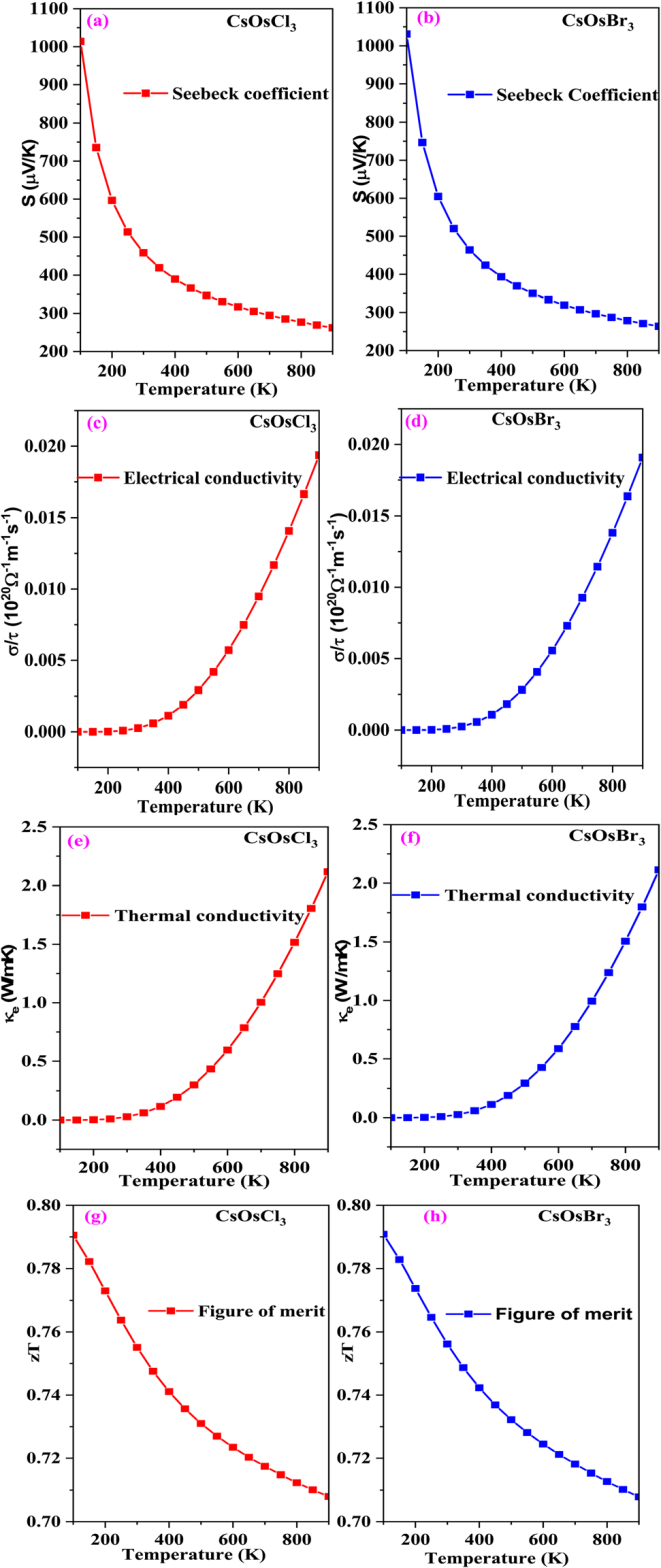
(a–h) The variation of Seebeck (a and b), electrical conductivity (c and d), thermal conductivity (e and f) and figure of merit (g and h) with temperature for CsOsX_3_ (X = Cl, Br) alloys.

At first, we have computed Seebeck coefficient. Its variation with temperature is shown in Fig. 6(a) and (b). From the graph we can see that as temperature increases its value decreases (from 1013.85 to 262.28 and from 1030.91 to 264.04) μV K^−1^ for CsOsCl_3_ and CsOsBr_3_ alloys respectively. The reason of this behaviour is as the temperature rises, more thermal energy is available to excite charge carriers from the valence band to the conduction band, leading to an increase in the concentration of free electrons and holes. This increased carrier concentration tends to diminish the Seebeck coefficient.^33^ The value of Seebeck is positive in entire temperature range indicating that holes are the majority charge carriers.

Next, we have computed electrical conductivity for both the materials. The variation with temperature is shown in Fig. 6(a) and (d). It increases with rise in temperature (from 0.0001 to 0.018 and from 0.0001 to 0.019) × 10^20^ Ω^−1^ m^−1^ s^−1^ for CsOsCl_3_ and CsOsBr_3_ alloys respectively. It depends on number of charge carriers as number of charge carrier increases with increase in temperature so electrical conductivity also increases.

We have also calculated thermal conductivity which consists of electronic and lattice both parts. Here, for these alloys we have only calculated the electronic component and excluded the lattice vibrations because BoltzTrap code is not capable to calculate the lattice part. The temperature dependency of electronic thermal conductivity is shown in Fig. 6(e) and (f).

Also, we have computed figure of merit (*zT*) which serves as a crucial measure in assessing the thermoelectric performance of materials. A value around or exceeding one indicates promising potential for thermoelectric device utilization. In Fig. 6(g) and (h), the relationship between the thermoelectric figure of merit (*zT*) and temperature is depicted. The value of *zT* at room temperature is 0.75 and 0.76 for CsOsCl_3_ and CsOsBr_3_ alloys respectively. The notably high *zT* values advocate for the potential applications of these materials in renewable energy and thermoelectric devices.

## 4. Conclusion

To summarize, we conducted first principles calculations on two halide perovskites to explore their stability considerations and potential applications in thermoelectric field. These materials exhibit structural phase stability in the *Pm*3̄*m* phase, which was confirmed through tolerance factor evaluations and structural optimization simulations. Mechanical stability is ensured by meeting the Born–Huang stability criteria, while both Pugh's ratio and Cauchy's pressure coefficients support the ductile nature of these perovskites. The examination of the electronic properties reveals that these compounds showcase a direct bandgap at the *Γ* symmetry points, with values of (1.36 and 1.10) eV for CsOsCl_3_ and CsOsBr_3_ alloys respectively. This characteristic makes them highly suitable for energy harvesting applications. Moreover, we have also studied the thermodynamic properties in 0–300 K temperature range. Also, these alloys have good value of *zT* which indicates that these materials are well suited for utilization in thermoelectric applications.

## Data availability

The data would be available from the corresponding author on a reasonable request.

## Author contributions

All the calculations and write up of manuscript has been carried out by Mrs Sakshi Gautam. The manuscript was check and hence modified by Dr Dinesh C. Gupta.

## Conflicts of interest

The authors have no conflict of interest.
